# Exploiting Kinetic Features of ORAC Assay for Evaluation of Radical Scavenging Capacity

**DOI:** 10.3390/antiox12020505

**Published:** 2023-02-17

**Authors:** Joana R. B. Carvalho, Andreia N. Meireles, Sara S. Marques, Bruno J. R. Gregório, Inês I. Ramos, Eduarda M. P. Silva, Luisa Barreiros, Marcela A. Segundo

**Affiliations:** 1 LAQV, REQUIMTE, Department of Chemical Sciences, Faculty of Pharmacy, University of Porto, R Jorge Viterbo Ferreira 228, 4050-313 Porto, Portugal; 2 TOXRUN—Toxicology Research Unit, University Institute of Health Sciences, CESPU, CRL, 4585-116 Gandra, Portugal; 3 Escola Superior de Saúde, Instituto Politécnico do Porto, Rua Dr. António Bernardino de Almeida 400, 4200-072 Porto, Portugal

**Keywords:** ORAC, data analysis, area under curve, lag time, kinetics, antioxidant capacity

## Abstract

The analysis and interpretation of data retrieved from Oxygen Radical Absorbance Capacity (ORAC) assays represent a challenging task. ORAC indexes originate from different mathematical approaches often lacking correct elucidation of kinetic features concerning radical scavenging reactions by antioxidant compounds. In this work, the expression of ORAC values as area under fluorescein (FL) decay curves (AUC) and lag time are critically compared. This multi-parametric analysis showed the extension of radical scavenging reactions beyond the lag time period for caffeic acid, gallic acid, reduced glutathione and quercetin, extending their antioxidant protection of FL. Ethanol delayed the reaction of both FL and antioxidant compounds with free radical species generated from 2,2′-azobis(2-amidinopropane) dihydrochloride thermolysis. Trolox equivalent values, commonly used to express ORAC values, were more affected by the differences in radical scavenging kinetics between the reference and the tested antioxidant compounds when calculated from AUC than from lag time. These findings stressed the importance of choosing calibrator compounds presenting ORAC kinetics similar to samples to prevent biased estimation of the antioxidant capacity. Additionally, the framework proposed here provides a sustainable analytical method for the evaluation of antioxidant capacity, with an AGREE score of 0.73.

## 1. Introduction

The meaning of Oxygen Radical Absorbance Capacity (ORAC) indexes as indicators of antioxidant capacity is currently under debate [1,2,3,4,5]. These assays entail the measurement of the protection afforded by an antioxidant compound to a target molecule (fluorescein (FL), for example) against oxidation by biologically relevant oxygen radical species (reactive oxygen species, ROS) [6]. It is still considered a valuable assay for the comparative evaluation of antioxidant scavenging ability as it gathers important analytical advantages [7,8]. ORAC is one of the most widely used methods for estimating total antioxidant capacity. It has the benefit of being a straightforward and standardized procedure, based on the generation of peroxyl radicals that are relevant in food and biological systems. However, the assay may be influenced by the existence of secondary reactions, reactions related to radical chain breaking processes, and the presence of metal ions [9,10]. The implementation is also easy in the laboratory, requiring a spectrophotometer or a fluorimeter depending on the chosen reporter probe. Further, among the points under discussion, data analysis along with the expression and interpretation of ORAC values should be highlighted [1,2,11]. Methodological aspects concerning assay conditions are also variable among studies, often hampering data comparison.

The antioxidant activity of a given sample results from the action of fast and slow reacting compounds towards free radicals and depends not only on the amount of antioxidant species (stoichiometry) but also on their reactivity against those radical species (rate of reaction) [12,13]. In addition, the antioxidant activity is often affected by the formation of by-products from the reaction between antioxidants and radicals, which can have antioxidant activity. These by-products may react at similar or slower rates compared to their parent compounds. Thus, the formation of slower reacting by-products is particularly noticeable when using fluorescent probes with low reactivity towards ROS, namely the classical probe used in ORAC assays, fluorescein. Food samples, for instance, present complex reaction kinetics towards ROS radicals, resulting from the mixture of multiple compounds. Hence, the prediction of ORAC curves shape for complex samples is not straightforward.

The two main methods most commonly applied to calculate ORAC values and evaluate the radical scavenging effects of antioxidants are namely lag time assessment [14,15,16,17] and net area under curve (net AUC) calculation [18,19,20,21,22]. Both methods for data analysis have been used in several studies, affording complementary information on the elucidation of antioxidant interaction with 2,2′-azobis(2-amidinopropane) dihydrochloride (AAPH) derived radicals using pyranine and FL as target molecules [13,23,24,25]. Lag time is evaluated as the period when the reaction between radical species and antioxidant compounds occurs before the decay of a target molecule (e.g., FL), which also works as a probe for reaction monitoring [13]. AUC values are calculated for probe fluorescence decay curves in the absence and in presence of antioxidant species. Net AUC corresponds to the increase in area afforded by the protection by antioxidant species with respect to the blank that corresponds to the maximum probe decay rate attainable for the set of reaction conditions chosen.

Generally, values are then expressed as Trolox equivalents (TE), the standard antioxidant compound [1,11]. Hence, ORAC values must be interpreted according to the parameter considered. ORAC indexes that result from AUC values integrate in a single value both in the extent and the initial time of radical scavenging reactions carried out by antioxidant molecules [1,26]. Nevertheless, without the respective curves, those values do not indicate the nature of the antioxidants present in complex extracts, neither refer to the kinetics of radical scavenging. More specifically, AUC values do not distinguish kinetic from stoichiometric aspects of antioxidant action [27]. Evaluation of lag time, on the other hand, clearly defines the amount of antioxidant compounds presenting radical scavenging rates that are considerably higher than those of target molecules. However, the lag time is not always present, and it is difficult to assess samples rich in slow reactants. Lag times do not account for effects that extend beyond the early stages of oxidation, such as the potential effects of antioxidant by-products and slow antioxidant reactions [11]. Moreover, variations in the calculation of ORAC values, namely at defining the initial point of reaction, delimiting the lag time and setting the time interval for AUC assessment, affect both approaches and yield incoherency among studies [5].

Another issue with ORAC methodologies is the variability of reaction conditions employed among different studies and the lack of standardized procedures. ORAC values are strongly dependent on the solubility of the compounds in the reaction media [28]. For that reason, the content in an organic solvent (dimethyl sulfoxide, methanol, or ethanol, often used for antioxidant extraction from complex samples) influences reaction kinetics and the availability of the compounds to react with the radicals [17,23,28]. For instance, the ORAC-FL values listed for quercetin range from 4.38 to 10.7, showing the impact of reaction conditions and sample preparation [29,30,31,32,33].

In this context, this work aimed at the critical evaluation of ORAC data analysis and interpretation when FL is used as a target molecule using a microchemical, sustainable approach, and at disclosing their meaning as antioxidant capacity indicators. For this purpose, several antioxidant compounds with different scavenging kinetics towards AAPH-derived radicals were submitted to ORAC-FL assays. The variation in the intrinsic fluorescence of the target molecule FL induced by oxygen radical species derived from the generator AAPH over time was monitored. Hence, FL works simultaneously as a target molecule and probe for reaction follow-up. When no antioxidant species is present, a fast decay in probe fluorescence intensity is noted. Generally, antioxidant compounds delay FL oxidation and consequently fluorescence in a concentration-dependent manner [1]. The information retrieved by the ORAC curves was disentangled into three main parameters: AUC, lag time and FL decay rate. The influence of ethanol (as a model for organic solvent effect) in these parameters was also evaluated. Finally, kinetic aspects concerning radical scavenging processes were taken into account to discuss the ORAC values assessed for model antioxidant compounds and complex food samples, namely from red wines and orange juices.

## 2. Materials and Methods

### 2.1. Reagents and Solutions

All chemicals used were of analytical reagent grade with no further purification. Water from Arium water purification systems (resistivity > 18 MΩ cm, Sartorius, Göttingen, Germany) was used for the preparation of all solutions. Absolute ethanol (Panreac, Barcelona, Spain) was employed for the preparation of the caffeic acid, gallic acid and quercetin stock solutions.

(±)-6-Hydroxy-2,5,7,8-tetramethylchromane-2-carboxylic acid (Trolox), 2,2′-azobis(2-amidinopropane) dihydrochloride (AAPH), caffeic acid, ascorbic acid, quercetin dihydrate and potassium phosphate monobasic were purchased from Sigma-Aldrich (St. Louis, MO, USA). Reduced glutathione (GSH) was supplied by Aldrich (Milwaukee, WI, USA). Fluorescein sodium (FL) and gallic acid were acquired from Fluka (Buchs, Switzerland).

Phosphate buffer (pH 7.4; 75 mM) was prepared by dissolving the respective solid in water to be used for preparation of all solutions after pH adjustment using either concentrated HCl or NaOH. Antioxidants and AAPH solutions were prepared daily. FL stock solution (0.58 mM) prepared in a phosphate buffer (pH 7.4; 75 mM) was stored in the dark at 4 °C and used to prepare FL working solutions within a week. On the day of analysis, this solution was diluted to a final concentration of 58.5 nM (working solution) immediately before use. An AAPH solution (40 mM) was also prepared in phosphate buffer (previously incubated at 37 °C) immediately before being added to the reaction mixture. Trolox (0.200 mM), GSH (0.200 mM) and gallic acid (2 mM) stock solutions were prepared by dissolving the respective solids in a phosphate buffer, whereas the ascorbic acid (100 mM) stock solution was prepared in water. Working standard solutions were prepared by rigorous dilution of the respective stock solutions in a phosphate buffer (pH 7.4; 75 mM). Concentration ranges of 2.0–10 μM were used for Trolox and gallic acid, whereas GSH and ascorbic acid were prepared at 2.0–8.0 μM and 3.5–10 μM, respectively. Quercetin (0.100 mM) and caffeic acid (0.500 mM) stock solutions were prepared in an ethanol–phosphate buffer (50:50, *v*/*v*). Standard solutions with concentrations ranging from 4 to 25 μM were prepared using the same solvent. The final ethanol concentration was adjusted to 5% (*v*/*v*) upon addition of the antioxidant solutions to microplate wells. Control wells were prepared likewise. Concentration of quercetin and caffeic acid in each well was between 0.40 and 2.5 μM. For comparison purposes, Trolox was also prepared in an ethanol–phosphate buffer (50:50, *v*/*v*), following the same preparation procedure described for quercetin and caffeic acid.

Orange juice and red wine samples (n = 5) were purchased at local supermarkets. Samples were diluted at two levels in a phosphate buffer (pH 7.4; 75 mM). Orange juice samples were diluted 100 to 1000 times, whereas wine samples were diluted 500 to 1500 times. Each dilution was analysed in triplicate.

### 2.2. ORAC-FL Procedure under Microplate Format

An ORAC-FL assay was conducted under a 96-well microplate format (Figure S1, Supplementary Data), setting the final volume of reaction mixture at 200 μL. Briefly, 120 μL of FL solution (well concentration at 35 nM) and 20 μL of antioxidant, sample or buffer solution were added to each well. The mixture was then incubated at 37 °C for 15 min with the subsequent addition of 60 μL of AAPH solution (well concentration at 12 mM). After homogenization, fluorescence intensity was recorded every min up to 3 h (λ_excitation_ = 485 nm; λ_emission_ = 520 nm). Control assays (n = 6) were performed, replacing the antioxidant solution by phosphate buffer (pH 7.4; 75 mM). Oxidation of FL in the absence of a radical source was also evaluated by replacing AAPH solution with a buffer solution. Solutions containing antioxidants were analysed in triplicate and on two independent days. The wells placed in the edges of the microplate were filled with 200 μL of water to ensure correct temperature control throughout the experiments. All fluorescence measurements were carried out at 37 °C using a Cytation3^®^ microplate reader (Bio-Tek Instruments, Winoosky, VT, USA) with fluorimetric detection, controlled by Gen5 (Bio-Tek Instruments) software.

### 2.3. Calculation of ORAC-FL Parameters to Assess Antioxidant Capacity

The reactions that describe the competition towards AAPH-derived radicals mainly represented by peroxyl radicals (ROO^•^) are depicted in the scheme below:

(1) $${\mathrm{ROO}}^{•}+\mathrm{XH}\overset{K1}{\to }X^{•}+\mathrm{ROOH}$$

(2) $${\mathrm{ROO}}^{•}+\mathrm{FL}\overset{K2}{\to }\mathrm{oxidized}\;\mathrm{FL}+\mathrm{ROOH}$$

(3) $$2{\;\mathrm{ROO}}^{•}\to \mathrm{non}\;\mathrm{radical}\;\mathrm{products}$$

**Figure 1:**
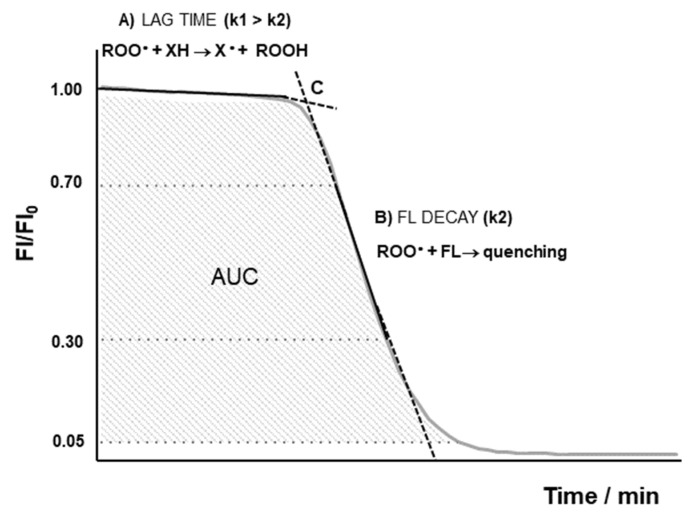
Schematic representation of relative fluorescence vs. time in ORAC assay. Segment A corresponds to reaction between fast reacting antioxidants and AAPH-derived radicals. Segment B (and adjacent zones) corresponds to fluorescein (FL) consumption by AAPH-derived radicals. Point C corresponds to the intersection of lines based on segments A and B.

As described elsewhere, FL is a slow scavenger of AAPH-derived radicals when compared to antioxidant compounds [34,35]. Therefore, the expected analytical signal corresponds to a lag time because of the consumption of the antioxidants present in the media (non-detectable probe oxidation) by the radical species (Equation (1)), followed by immediate decay in the FL probe fluorescence caused by radical-mediated oxidation (Equation (2)). A typical ORAC profile or FL decay curve (fluorescence intensity versus time) is depicted in Figure 1.

Fluorescence decay curves were first normalized in relation to initial fluorescence intensity values (FI/FI_0_). Then, AUC was calculated up to values corresponding to 5% of the initial fluorescence value. This limit was set for the standardization of AUC calculation, avoiding bias due to the contribution of the tail of the curve.

Lag time was graphically assessed by the intersection (Figure 1, point C) between the line corresponding to minimal probe decay (Figure 1, curve A) and the line corresponding to maximum probe decay (Figure 1, curve B), established between 30 and 70% of probe decay. The slope of curve B value accounts for the fluorescence decay rate after AAPH-derived radicals had depleted all antioxidant species, which are faster radical scavengers than FL. The relation between lag time and antioxidant concentration ([XH] in μM) can be expressed as *lag time* = *n*[XH]/*R_i_*, where *n* corresponds to the stoichiometric number for antioxidants and *R_i_* corresponds to the radical generation rate upon AAPH thermolysis.

For data comparison purposes, the antioxidant activity of all compounds under study was converted into Trolox equivalents (TE) by dividing the slopes of the respective calibration curve by the slope of the calibration curve obtained for the reference antioxidant compound Trolox [36,37]. A paired *t*-test was performed to compare slope and intercept values of the calibration curves’ AUC/lag time versus antioxidant concentration.

## 3. Results

### 3.1. Lag Time Parameter

**Figure 2:**
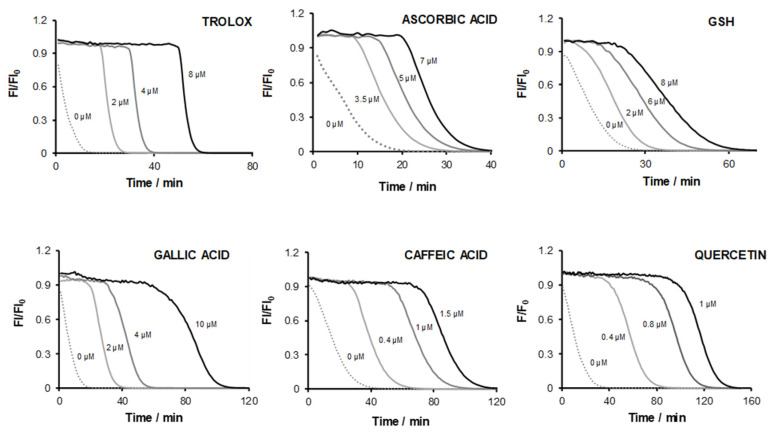
ORAC curves obtained for Trolox, ascorbic acid, reduced glutathione (GSH), gallic acid, caffeic acid and quercetin.

Fluorescein decay induced by AAPH-derived radicals was monitored in the presence of increasing concentrations of Trolox, ascorbic acid, gallic acid, GSH, caffeic acid and quercetin (chemical structures available in Figure S2, Supplementary Data). Fluorescence decay curves are depicted in Figure 2.

A linear relation between lag time and antioxidant concentration was established for all antioxidants. AUC values were also plotted against antioxidant concentration. The main analytical parameters calculated from both plots are compiled in Table 1.

### 3.2. Probe Fluorescence Decay Rate

Considering that an excess of AAPH was added to the reaction media, free radicals are generated at a constant rate [38] and, therefore, pseudo-first order conditions are achieved for this reagent. FL decay rate, which is directly related to the decrease in its intrinsic fluorescence, was also estimated for ORAC curves attained for the model antioxidant compounds under study. As a control, FL stability was also evaluated in the absence of both radical and antioxidant species and the differences in fluorescence values were <12% after 4 h. Therefore, FL autoxidation was negligible within this timespan. In aqueous media, FL decay rates in the presence of Trolox (Table 2) and ascorbic acid (0.085–0.095 min^−1^) were similar, whereas for the remaining compounds, the values were generally lower (<0.065 min^−1^). To verify the formation of oxidation by-products capable of prolonging antioxidant action beyond lag time periods, a paired *t*-test was performed to compare FL decay rates obtained for each concentration of antioxidant compounds and the control (absence of antioxidant). No statistically significant differences were observed for Trolox, ascorbic acid and GSH, presenting |*t*|_calculated_ values < 1.995 (*t*_tabulated (*P* = 0.05; d.f. = 7)_ = 2.776). For those compounds, the FL decay rate did not vary significantly with antioxidant concentration within the tested range.

For caffeic acid, quercetin and gallic acid, evidence of the accumulation of oxidation by-products for concentrations above 0.8, 2,and 4 μM, respectively, was found. For instance, the |*t*|_calculated_ value for caffeic acid was 4.082, confirming that the decay rate was significantly lower with respect to the control for concentrations above 0.8 μM (*t*_tabulated (*P* = 0.05; d.f. = 7)_ = 2.776). A similar result was obtained for gallic acid above 4 μM and quercetin above 2 μM with |*t*|_calculated_ values > 3.12 (*t*_tabulated (*P* = 0.05; d.f. = 7)_ = 2.776). In these cases, FL decay rates reflected the simultaneous scavenging of radicals by FL and secondary antioxidant species at similar rates. For lower concentrations, |*t*|_calculated_ values were <2.66 (*t*_tabulated (*P* = 0.05; d.f. = 7)_ = 2.776), showing no statistically significant difference.

### 3.3. Effect of Ethanol in Parameter Assessment

The effect of organic solvent in the reaction media on ORAC parameters was evaluated using Trolox as model antioxidant and ethanol as model organic solvent. For this purpose, ORAC curves for Trolox were established in a phosphate buffer and in a ethanol-phosphate buffer (5:95, *v*/*v*). Lag time, AUC and FL decay rates are presented in Table 2.

Additionally, the generation rate of AAPH-derived radicals in both media was calculated. Values were 6.02 × 10^−3^ and 5.72 × 10^−3^ μM s^−1^ in the phosphate buffer and in the ethanol–phosphate buffer (5:95, *v*/*v*), respectively.

For the ethanol-aqueous media, calibration curves for Trolox were defined as [Trolox] (μM) = 5.83 (±0.46) Lag time (min) + 21.66 (±1.20) and [Trolox] (μM) = 5.97 (±0.45) AUC (min) + 30.49 (±3.01), respectively. Slopes obtained for the phosphate buffer media are presented in Table 1 (5.51 (±0.18) and 5.25 (±0.18) for lag time and AUC, respectively). Intercept values were 10.65 ± 1.19 and 15.37 ± 1.20 min for lag time and AUC, respectively. The paired *t*-test was performed to compare slope and intercept values. For lag time curves, |*t*|_calculated_ values were 1.71 and 8.82 (*t*_tabulated (*P* = 0.05; d.f. = 7)_ = 2.31), respectively. These values indicate no statistically significant difference between the slopes, but a significant increase in the intercept value for the ethanol-aqueous media. For AUC curves, |*t*|_calculated_ values were 3.93 and 12.35 (*t*_tabulated (*P* = 0.05; d.f. = 7)_ = 2.31) for the slope and intercept, respectively, indicating a statistically significant difference for both parameters. For all conditions studied, intercept values were statistically different from AUC, and lag time values attained for the blank as|*t*|_calculated_ values were <18.81 and >26.80 (*t*_tabulated (*P* = 0.05; d.f. = 7)_ = 2.45) for lag time and AUC, respectively.

### 3.4. Antioxidant Capacity Reported as Trolox Equivalents (TE)

For all the compounds studied, ORAC values calculated from lag time and AUC data were converted into Trolox equivalents (TE) (Table 3). TE values previously reported for those compounds were also collected from the literature and compiled in Table 3 for comparison purposes.

In general, TE values obtained from lag time assessment were lower than those based on AUC values (|*t*|_calculated_ > 4.08; *t*_tabulated (*P* = 0.05; d.f. = 10)_ = 2.23), except for ascorbic acid and quercetin. For these compounds, |*t*|_calculated_ values were <1.93 (*t*_tabulated (*P* = 0.05; d.f. = 10)_ = 2.23), and thus no statistically significant differences were observed between TE_AUC_ and TE_LT_ values.

### 3.5. Analysis of Antioxidant-Containing Beverages

**Figure 3:**
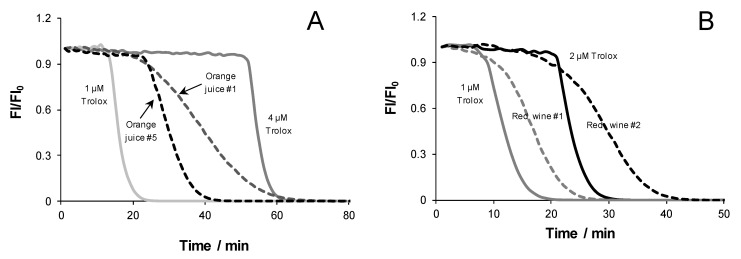
ORAC curves obtained for the standard antioxidant Trolox (full lines), juice samples (**A**, dashed lines) and wine samples (**B**, dashed lines).

The influence of the rationale applied upon data analysis was evaluated for commercial orange juice (n = 5) and red wine samples (n = 5) (Figure 3).

Among the orange juice samples, profiles showing the presence of both slow and fast reacting compounds were obtained (Figure 3A). All red wine samples yielded profiles indicating the presence of slow reacting compounds (Figure 3B).

The repercussions of profile differences in ORAC values were assessed by calculating TE values from both lag time and AUC values (Table 4).

The parameters of AUC and lag time were influenced by the nature of the antioxidants present in the samples. For instance, for juice samples 1 to 4, TE values calculated from the AUC curves were 53% to 97% higher than those based on the lag time assessment. On other hand, in juice sample 5, this difference was only 24%. Accordingly, the same sample presented the highest FL decay rate among all samples tested for the same level of dilution (data not shown), and the value was similar to those obtained when Trolox or ascorbic acid were the only antioxidants in the reaction media.

Regarding red wine samples, TE_AUC_ values were 19% to 63% higher than their TE_LT_ counterparts. In this set of samples, the red wine 5 sample presented the lowest difference between TE_AUC_ and TE_LT_.

### 3.6. Analysis of AGREE Metric

The AGREE score was determined with the Analytical GREEnness calculator. The final evaluation score resulted from the product of the assessment of the 12 principles of Green Analytical Chemistry [42]. This value was calculated for the ORAC-FL assay format proposed here, providing a value of 0.73 (Figure S3A), with a default weight of two on a scale of four for all the evaluation criteria.

## 4. Discussion

The shape of fluorescence ORAC decay curves (Figure 2), in increasing concentrations of ascorbic acid and Trolox, presented a clear distinction between lag time and FL decay periods. For the remaining antioxidants, this distinction was not always as clear, denoting more complex scavenging kinetics. For all compounds, the occurrence of a plateau along the initial fluorescence measurement confirmed that those compounds were faster reactants towards AAPH-derived radicals compared to FL.

For Trolox and ascorbic acid, method sensitivity, given by the slope of the calibration curves (min μM^−1^), was similar for both the AUC and lag time approaches (Table 1). However, for GSH, quercetin, caffeic acid and gallic acid, this value was higher using AUC compared to lag time. For both parameters, a loss in linearity was observed for caffeic acid, quercetin and gallic acid using concentrations above 1.0, 2.0 and 10 μM, respectively.

For quercetin and caffeic acid, the shape of the ORAC curves remained unchanged, even at the highest tested concentrations with no evidence of the formation of oxidation by-products. Interestingly, the difference between slope _LT_ and slope _AUC_ (Table 1) was higher for caffeic acid (ca. 46%) than quercetin (ca. 12%), showing that the last is a faster scavenger of free radical species, which might be due to either higher reactivity or stoichiometry capacity towards those species. On the contrary, a rounding effect in the decay part was particularly noticeable for gallic acid at 10 μM (Figure 2). This result supports the accumulation of by-products of radical-scavenging reactions by antioxidants that react with radical species at a rate similar to that of FL. Therefore, antioxidant action was extended through slower kinetics. Consequently, lag times become more difficult to define and thus ORAC values based on this parameter are less accurate. Under such conditions, it was not possible to isolate the protection afforded by antioxidant molecules in the original redox status from the action of by-products generated upon radical scavenging.

The validity of the comparison of net AUC values relies on the addition of AAPH solution to microplate wells. Reproducibility of reaction conditions, namely temperature control and reagents mixing in the plate, is critical and highly conditioned by operator skills. Hence, the impact of premixing well content after AAPH addition by multichannel pipetting was evaluated. It was observed that initial fluorescence values obtained for control curves (wells with both FL and AAPH, no antioxidant) were 8 to 15% lower than those obtained in the presence of antioxidant compounds for an AAPH/FL molar ratio of 3.4 × 10^5^ (Figure 2). When no antioxidant is added to the reaction media, FL oxidation mediated by radical species starts before the acquisition of the first data points. Therefore, AUC values depend on the time taken between the addition and mixing of AAPH in microplate wells, and the acquisition of the first data points, having an impact on results when calculating net AUC. This effect can be seen in Figure 2, when comparing the control curves (0 µM) for Trolox and gallic acid to ascorbic acid, GSH and caffeic acid counterparts. It means that a longer time was taken between microplate preparation and fluorescence reading in the Trolox and gallic acid experiments.

Despite this effect, an adequate mixing of the reaction media upon AAPH addition must not be dismissed, especially when organic solvents are present in the reaction media. Halving the molar ratio of AAPH/FL (1.71 × 10^5^) or decreasing the time between AAPH addition and fluorescence reading (by eliminating the premixing step) reduced this difference to values < 5%, confirming this idea. These findings denote the urge for standardization of this critical step, increasing inter-day precision and enabling inter-laboratorial comparisons.

As indicated above, AUC values result from the combination between lag time periods and FL decay rates. After antioxidant depletion, FL is then oxidized independently from antioxidant concentration, leading to a consequent decrease in its intrinsic fluorescence. This decrease (Figure 1, curve B) reflects the rate and the extension of molecular interactions between AAPH-derived radicals and FL (Equation (2)).

For Trolox, ascorbic acid and GSH, the FL decay rate did not vary significantly with antioxidant concentration within the tested range (Figure 2). Hence, these antioxidant compounds were consumed by free radicals during the lag phase, showing a higher reaction rate towards AAPH-derived radicals compared to FL. After the lag phase period, fluorescence decay was independent from antioxidant initial concentration.

Considering caffeic acid, quercetin and gallic acid, the accumulation of oxidation by-products was found. In this case, FL decay rates reflected the simultaneous scavenging of radicals by FL and secondary antioxidant species at similar rates.

Lag time, AUC and FL decay parameters were affected by the addition of ethanol to the reaction media (Table 2). Lag time and AUC values were higher in the ethanol-aqueous media with respect to the aqueous buffer, which means that the reaction between Trolox and AAPH-derived radicals was delayed in the organic media. The effect of organic solvents in antioxidant mechanisms has been reported before [43,44,45,46]. In fact, ORAC values tend to be lower with decreasing reaction media polarity regardless of the probe, which is attributed either to solubility enhancement (e.g., quercetin and caffeic acid) or to changes in the H-atom donor abilities of compounds [44]. In our experiments, AUC values calculated for control curves were also doubled in the presence of ethanol, suggesting the reaction between FL and radical species was delayed as well (Equation (2)). Considering the many prototropic forms of fluorescein, one possible explanation might be that the deprotonation of reactive hydroxyl groups of FL (Figure S2, Supplementary Data) was favored in the aqueous media with respect to the ethanol-aqueous media [47]. This is the rate-limiting step for the interaction between FL and AAPH-derived radicals [34]. Additionally, the generation rate of AAPH-derived radicals in both media was calculated and radical generation was not significantly affected by the presence of ethanol at 5% (*v*/*v*).

The influence of ethanol in method performance was evaluated through calibration curves of lag time or AUC against Trolox concentration. For all conditions studied, intercept values were statistically different from AUC and lag time values attained for the blank. It would be expected that intercept values for lag time curves would be close to zero because, in the absence of antioxidant species, FL oxidation induced by radical species is theoretically immediate, fostering null values for lag time. However, intercept values were at least 10 min. This effect was particularly visible for ethanol-aqueous media as intercept values were doubled with respect to aqueous media, reinforcing that the rate of FL oxidation was decreased by ethanol.

In general, the TE values obtained from the lag time assessment were lower than those based on AUC values (Table 3), except for ascorbic acid and quercetin. For these compounds, no statistically significant differences were observed between TE_AUC_ and TE_LT_ values. TE_AUC_ values were more affected by the differences in radical scavenging kinetics between the reference antioxidant (Trolox) and the working antioxidant compound compared to TE_LT_. Overestimation of ORAC values arising from AUC values were due to the differences in probe decay rate, which did not affect lag time periods. For instance, GSH and caffeic acid yielded a ca. 44% increase in TE values from those that were calculated from AUC values compared to lag time, which confirms that the kinetics of free-radicals scavenging was slower from that of Trolox as the antioxidant action extended beyond lag time periods. On the other hand, for ascorbic acid, TE_AUC_ and TE_LT_ were equal, which means that the scavenging kinetics were equivalent to Trolox. These findings also support the choice of calibrator compounds presenting similar ORAC kinetics to avoid over or underestimation of TE values, as previously described for other methods of antioxidant capacity assessment [36,37].

Based on TE values, compounds can be ranked as GSH < ascorbic acid < gallic acid < caffeic acid < quercetin, which agrees with previous works (Table 3). However, a wide range of ORAC values has been reported for these compounds, reflecting differences in assay conditions [48] or data analysis and reporting among studies [1]. Therefore, it is not possible to make any comparison of absolute ORAC values. Quercetin and caffeic acid showed higher antioxidant capacity values when compared to the previous literature reports (Table 3). It has already been described that differences in the content and nature of organic solvents (e.g., acetone, methanol, ethanol, dimethyl sulfoxide) change antioxidant solubility and, thus the ORAC values. Another factor for variability is that the reactivity of antioxidants towards radicals and FL spectroscopic properties varies by pH [48,49].

Independently from the parameter (LT or AUC), the ORAC values obtained for orange juice samples (Figure 3A) were lower than those of red wines (Figure 3B, Table 4), which is in agreement with the literature reports [50,51]. Furthermore, no dilution effect was observed within the range applied, indicating that TE_AUC_ and TE_LT_ were independent of dilution factors for all samples.

The nature of the antioxidants present in the samples had a greater influence on AUC than in on lag time. In the case of juice sample 5, the effect was not so significant, indicating that fast radical scavengers (e.g., ascorbic acid) made a greater contribution to the overall antioxidant capacity when compared to slower scavengers.

Relative to the red wine samples, TE_AUC_ were higher than TE_LT_, showing different contents in slow radical scavengers. This might be because wine antioxidants work mostly as slow reactants towards peroxyl radicals (e.g., gallic and caffeic acid) in contrast with the standard compound Trolox. For wine sample 5, the role of slow scavengers and/or oxidation by-products was less pronounced when compared to the remaining samples, considering that red wine 5 presented the lowest difference between TE_AUC_ and TE_LT_.

Given the above, the ratio of slow to fast radical scavengers of a given sample is a critical parameter to be taken into consideration upon the selection of an appropriate calibrator compound. Similarly, in relation to pure antioxidant compounds, ORAC profiles of complex samples might not match the standard compound, leading to over or underestimation of ORAC values.

In terms of sustainability, the ORAC-FL assay format proposed here presented an AGREE score of 0.73 (Figure S3A). To improve this value (target 1.0), the proposed procedure could foster direct analysis by decreasing the sample volume up to a few microlitres. Furthermore, the use of non-bio-based reagents decreased the performance of some metric parameters. Still, compared to the fluorimetric analysis performed using conventional equipment [6] (AGREE score of 0.59) (Figure S3B), the present method offers the possibility of simultaneous analysis of up to 240 samples per hour with effluent production in the 200-microliter range per sample, contributing to the implementation of Green Analytical Chemistry.

## 5. Conclusions

The present approach extended the knowledge on ORAC methodological aspects, which contributed to the interpretation of data arising from the analysis of compounds frequently present in biological and food samples. This study provides a better understanding of the significance of ORAC values and, consequently, of the dynamics of radical scavenging mechanisms mediated by antioxidants, expanding the focus of ORAC assays beyond screening. The present strategy of ORAC data analysis pursues the clarification of the meaning of ORAC values, which comprises a step forward towards the inter-laboratory comparison of antioxidant capacity. For that, complementary information provided by different rationales, namely through the assessment and evaluation of AUC values, lag time values and FL decay rates must be gathered. In practice, the kinetic and stoichiometric components of antioxidant activity cannot be separated using AUC values. However, this approach combines the extent and the initial time length of antioxidant molecule-mediated radical scavenging processes into a single measurement. Conversely, the evaluation of lag time indicates the quantity of antioxidant compounds that demonstrate radical scavenging behavior. Determining lag time values becomes less specific in the presence of antioxidant action with slower kinetics, hence, it was not possible to separate the protection provided by antioxidant molecules in their direct scavenging activity from the effects of the by-products produced during radical scavenging. Considering what was mentioned above, lag time determination is recommended for pure and fast radical scavenging compounds and, for other conditions such as complex natural samples, AUC is recommended.

The integrated analysis of all three parameters supports a better understanding of the role of antioxidant compounds with different natures in complex samples. For instance, the comparison of FL decay rates beyond lag time periods leads to a better visualization of the formation of by-products upon radical scavenging by antioxidants, which carry on their protective effect. Although substantial data sets are created, particularly when analysing a large number of samples, automated data treatment using the current software can be implemented.

The establishment of standard procedures for the preparation of antioxidant-containing samples or standards is in order. Antioxidant compounds presenting low solubility in assay buffers might lead to underestimations of ORAC values. This issue can be overcome by resorting to organic solvents to solubilize the compounds. However, ethanol content affects TE values as it delays the kinetics of the radical scavenging processes. For that reason, the amount of ethanol used for the preparation of standard solutions should meet the value expected for the samples after dilution to avoid overestimation of TE values.

Finally, this work contributes to the elaboration of standardized parameter calculations, including lag time, AUC and reaction rate, using commonly available computer programs that should be spread within the scientific community, contributing also to the implementation of sustainable Green Analytical Chemistry.

## Figures and Tables

**Table 1:** Slope of calibration curves ^a^ established using lag time (LT) and area under curve (AUC) vs. antioxidant (AO) concentration.

AO Compounds	Linear Range (μM)	Slope_LT_ (min μM^−1^)	Slope_AUC_ (min μM^−1^)
Trolox	2.0–10	5.51 ± 0.18	5.25 ± 0.18
Ascorbic acid	3.5–9.0	2.89 ± 0.06	2.78 ± 0.13
GSH	2.0–8.0	1.90 ± 0.11	2.56 ± 0.12
Gallic acid	2.0–10	6.16 ± 0.16	6.54 ± 0.21
Quercetin	0.40–2.0	88.30 ± 2.77	99.22 ± 2.48
Caffeic acid	0.40–1.0	33.63 ± 1.29	49.34 ± 1.81

^a^ Calibration curves were established for 5 standard solutions of each antioxidant compound. Standard solutions were analysed in triplicate on two independent days (R > 0.9964).

**Table 2:** Effect of ethanol content in AUC, lag time and FL decay rate values obtained for Trolox.

Phosphate Buffer				Phosphate Buffer:Ethanol (95:5, *v*/*v*)		
[Trolox] (μM)	AUC (min)	Lag Time (min)	FL Decay Rate (min^−1^)	AUC (min)	Lag Time (min)	FL Decay Rate (min^−1^)
0	5.9 ± 3.1	N/A	0.066 ± 0.016	12.2 ± 2.9	N/A	0.040 ± 0.004
2.0	24.7 ± 3.0	20.5 ± 2.8	0.115 ± 0.036	40.3 ± 2.1	30.5 ± 3.1	0.053 ± 0.011
4.0	37.2 ± 3.3	33.5 ± 3.7	0.123 ± 0.040	55.2 ± 2.9	46.7 ± 3.2	0.052 ± 0.012
6.0	47.8 ± 4.0	44.5 ± 5.0	0.121 ± 0.043	68.2 ± 3.8	58.9 ± 3.7	0.051 ± 0.014
8.0	57.7 ± 4.6	55.3 ± 6.5	0.114 ± 0.035	80.9 ± 4.7	69.7 ± 4.7	0.050 ± 0.016
10	66.9 ± 6.1	64.7 ± 7.8	0.121 ± 0.045	87.2 ± 5.5	77.2 ± 5.1	0.048 ± 0.017

N/A: not applicable.

**Table 3:** Trolox equivalents (TE) calculated from AUC and lag time (LT) values for standard antioxidant compounds.

AO Compounds	TE_AUC_	TE_LT_	TE Literature
Ascorbic acid	0.53 ± 0.03	0.52 ± 0.02	0.30–0.95 ^a–c^
GSH	0.49 ± 0.03	0.34 ± 0.02	0.62–0.65 ^a,d^
Gallic acid	1.25 ± 0.06	1.12 ± 0.05	0.90–1.7 ^c,e,f^
Quercetin	16.62 ± 1.32	15.15 ± 1.32	4.38–10.7 ^a,e–h^
Caffeic acid	8.26 ± 0.69	5.77 ± 0.69	2.70–6.63 ^a,c,h^
Trolox	1.0	1.0	1.0

^a^ Ou et al. [30]; ^b^ Takashima et al. [13]; ^c^ Nenadis et al. [39]; ^d^ Liao et al. [40]; ^e^ Ou et al. [31]; ^f^ Pérez et al. [41]; ^g^ Zhang et al. [33]; ^h^ Davalos et al. [29].

**Table 4:** ORAC values calculated from AUC and lag time (LT) values obtained for orange juice and red wine samples.

Sample	TE_AUC_^a^	TE_LT_^b^
Orange juice 1	1.16 ± 0.02	0.59 ± 0.04
Orange juice 2	0.52 ± 0.01	0.34 ± 0.01
Orange juice 3	1.02 ± 0.13	0.57 ± 0.02
Orange juice 4	0.54 ± 0.02	0.32 ± 0.01
Orange juice 5	0.36 ± 0.01	0.29 ± 0.01
Red wine 1	4.02 ± 0.32	2.98 ± 0.11
Red wine 2	3.81 ± 0.27	2.34 ± 0.25
Red wine 3	3.98 ± 0.29	2.54 ± 0.11
Red wine 4	4.03 ± 0.33	2.52 ± 0.13
Red wine 5	3.15 ± 0.35	2.64 ± 0.30

^a,b^ Mean Trolox equivalents (TE) ± standard deviation corresponding to two levels of sample dilution (100 to 1000 times and 500 to 1500 times for juice and red wine, respectively) given in μmol of Trolox per mL of beverage. RSD < 12.6%. For juice samples, RSD values were <12.6% and <12.4% for TE_AUC_ and TE_LT_, respectively. Concerning red wine samples, RSD values were <8.3% and <10.7% for TE_AUC_ and TE_LT_ values, respectively.

## Data Availability

The data presented in this study are available in the article and supplementary material.

## Supplementary Materials

The following supporting information can be downloaded at https://www.mdpi.com/article/10.3390/antiox12020505/s1, Figure S1: Representation of 96-well microplate organization for ORAC-FL assays; Figure S2: Chemical structures of fluorescein and antioxidant compounds under study; Figure S3: General result of AGREE assessment of ORAC-FL assay under 96-well microplate (A), ORAC-β-PE assay in fluorescence spectrophotometer (B) and color scale for reference (C).
