# Supplementary material for: Exploiting Kinetic Features of ORAC Assay for Evaluation of Radical Scavenging Capacity
Source: Antioxidants (Basel). 2023 Feb 17;12(2):505. doi: 10.3390/antiox12020505 (PMC9951910; doi:10.3390/antiox12020505)
Supplement: Supplementary file 1 [file antioxidants-12-00505-s001.zip › antioxidants-2214804-supplementary.pdf]

Electronic Supplementary Data

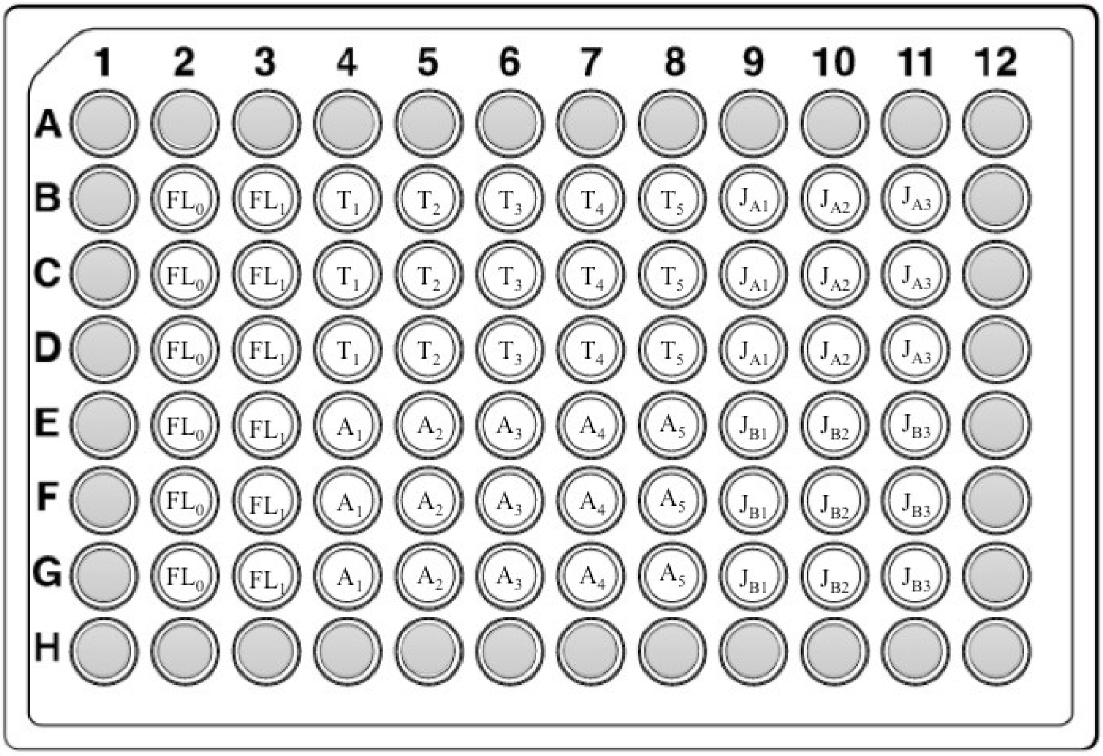

Fig. S1. Representation of 96-well microplate organization for ORAC-FL assays. Example: FL_0, 120 µL of fluorescein (35 nM) + 80 µl phosphate buffer (pH 7.4, 75 mM); FL_1,, 120 µL of fluorescein + 60 µL of AAPH (12 mM) + 20 µL phosphate buffer; T_1-T_5, 120 µL of fluorescein + 20 µL of Trolox with increasing concentrations + 60 µL of AAPH; A_1-A_5, 120 µL of fluorescein + 20 µL of ascorbic acid with increasing concentrations + 60 µL of AAPH; J_{A1}-J_{A3}, 120 µL of fluorescein + 20 µL of juice A dilution + 60 µL of AAPH; J_{B1}-J_{B3}, 120 µL of fluorescein + 20 µL of juice B dilution + 60 µL of AAPH. Edge wells were filled with 200 µL of water.

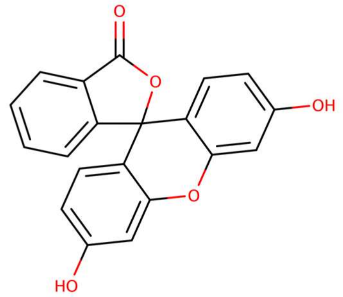

Fluorescein (FL)

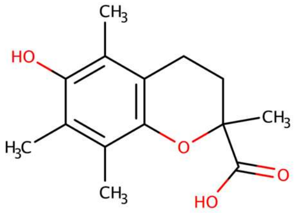

Trolox

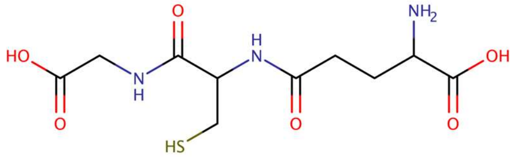

Reduced glutathione (GSH)

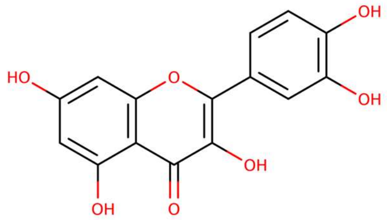

Quercetin

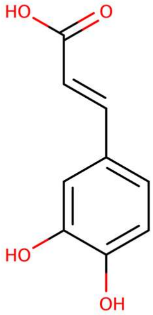

Caffeic acid

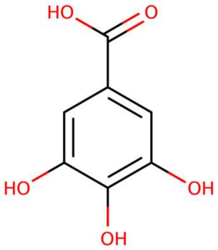

Gallic acid

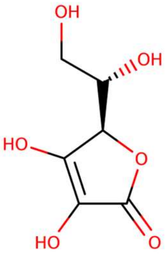

Ascorbic acid

Fig. S2. Chemical structures of fluorescein and antioxidant compounds under study.

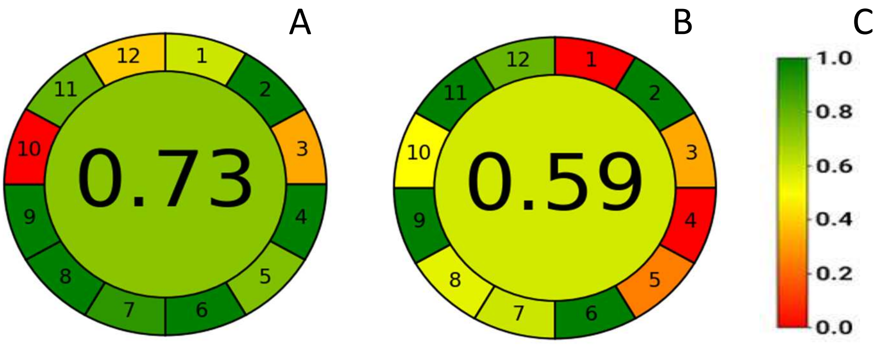

Fig. S3. General result of AGREE assessment of ORAC-FL assay under 96-well microplate (Fig. S3 A), ORAC-β-PE assay in fluorescence spectrophotometer (Fig. S3 B) and color scale for reference (Fig. S3 C). Parameter 1: Sample treatment; Parameter 2: Sample amount; Parameter 3: Device positioning; Parameter 4: Sample preparation stages; Parameter 5: Automation, miniaturization; Parameter 6: Derivatization; Parameter 7: Waste; Parameter 8: Analysis throughput; Parameter 9: Energy consumption; Parameter 10: Source of reagents; Parameter 11: Toxicity and Parameter 12: Operator´s safety.
